# A TET2 rs3733609 C/T genotype is associated with predisposition to the myeloproliferative neoplasms harboring JAK2^V617F^ and confers a proliferative potential on erythroid lineages

**DOI:** 10.18632/oncotarget.7072

**Published:** 2016-01-29

**Authors:** Xiao-hui Shen, Nan-nan Sun, Ya-fei Yin, Su-fang Liu, Xiao-liu Liu, Hong-ling Peng, Chong-wen Dai, Yun-xiao Xu, Ming-yang Deng, Yun-ya Luo, Wen-li Zheng, Guang-sen Zhang

**Affiliations:** ^1^ Division of Hematology, Institute of Molecular Hematology, The Second Xiang-Ya Hospital, Central South University, Changsha, Hunan 410011, P.R. China

**Keywords:** myeloproliferative neoplasms, TET2, rs3733609, clinical significance, mechanisms

## Abstract

Common germline single-nucleotide polymorphisms (SNPs) at JAK2 locus have been associated with Myeloproliferative neoplasms (MPN). And, the germline sequence variant rs2736100 C in TERT is related to risk of MPN, suggesting a complex association between SNPs and the pathogenesis of MPN. Our previous study (unpublished data) showed that there was a high frequency distribution in rs3733609 C/T genotype at Ten-Eleven Translocation 2 (TET2) locus in one Chinese familial primary myelofibrosis. In the present study, we evaluate the role and clinical significance of rs3733609 C/T genotype in JAK2V617F-positive sporadic MPN (*n* = 181). TET2 rs3733609 C/T genotype had a higher incidence (13.81%; 25/181) in JAK2V617F-positive sporadic MPN patients than that in normal controls (*n* = 236) (6.35%; 15/236), which was predisposing to MPN (odds ratio(OR) = 2.361; *P* = 0.01). MPN patients with rs3733609 C/T genotype had increased leukocyte and platelets counts, elevated hemoglobin concentration in comparison with T/T genotype. Thrombotic events were more common in MPN patients with rs3733609 C/T than those with T/T genotype (*P* < 0.01). We confirmed that rs3733609 C/T genotype downregulated TET2 mRNA transcription, and the mechanism may be involved in a disruption of the interaction between CCAAT/enhancer binding protein alpha (C/EBPA) and TET2 rs3733609 C/T locus.TET2 rs3733609 C/T genotype stimulated the erythroid hematopoiesis in MPN patients. Altogether, we found a novel hereditary susceptible factor-TET2 rs3733609 C/T variant for the development of MPN, suggesting the variant may be partially responsible for the pathogenesis and accumulation of MPN.

## INTRODUCTION

The most common BCR-ABL negative MPN includes polycythemia vera (PV), essential thrombocythemia (ET), and primary myelofibrosis (PMF) [1]. In the three main subtypes, mutually exclusive somatic mutations have been identified in about 90% of MPN cases [2]. JAK2V617F mutation is present in the majority of PV patients, and about half of ET and PMF cases [3–5], while MPL and CALR mutations are exclusively detected in ET and PMF patients, suggesting the presence of one or more genes variants is associated with risk of MPN [6–10]. Previously, a common haplotype (GGCC or 46/1) at the JAK2 locus has been confirmed to predispose to JAK2-positive sporadic and familial MPN [11–13]. Recently, the germline sequence variant rs2736100 C in TERT contributes to sporadic and familial MPN has also been reported [14, 15]. These germline variations at JAK2 and TERT loci may explain part of the population risk for developing MPN. However, the presence of host-modifying genetic factors or concomitant mutations in other genes, such as TET2, ASXL1, could modulate the MPN phenotype and contribute to the pathogenesis of MPN [16–18].

Loss-of-function mutations in TET2 are found in 5%–17% of MPN and have been shown to decrease 5-hmc levels [19–21]. TET2 is an important regulator for normal myelopoeisis. Murine models with disrupted TET2 display an expansion of the HSC compartment, myelomonocytic proliferation, and features resembling CMML [22]. TET2 mutations can also increase risk of leukemic transformation and shorten survival time in MPN patients [23].

Previously, we noted that the frequency of rs3733609 C/T genotype (located in TET2 exon 9)(http://www.ncbi.nlm.nih.gov/snp/?term = rs3733609) was much higher in one familial PMF than sporadic PMF (unpublished data), suggesting rs3733609 may be a MPN predisposition allele. In the present study, we attempted to determine whether the C allele of the rs3733609 is associated with the predisposition to sporadic MPN harboring JAK2V617F mutation and especially with the clinical phenotypes and the complications of MPN. Furthermore, we also evaluated the effects of the rs3733609 on myeloid lineage proliferation and explored in part the possible mechanism of rs3733609 C/T genotype in developing MPN.

## RESULTS

### The distribution of TET2 rs3733609 SNP in MPN patients

**Figure 1:**
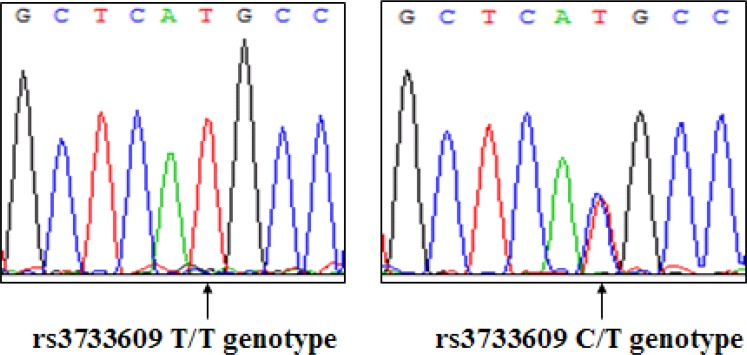
A representative DNA sequence of TET2 rs3733609 genotype (Left: T/T genotype; Right: C/T genotype)

To determine whether rs3733609 C/T genotype was associated with the predisposition to sporadic MPN, we analyzed the distribution of TET2 rs3733609 C/T genotype using PCR-direct sequencing in 181 MPN patients with JAK2V617F-positive and 236 healthy controls. A typical DNA sequencing of TET2 rs3733609 genotype was shown in Figure 1. Table 1 showed in detail the distribution of TET2 rs3733609 genotypes in MPN patients and controls. The rs3733609 C/T genotype existed in 25 of 181 MPN patients with JAK2V617F-positive (25/181; 13.81%), and the rest possessed the rs3733609 T/T genotype. The rs3733609 C/T genotype was also detected in 15 of 236 healthy controls (15/236; 6.35%). In order to avoid the impact of classic TET2 mutations on C/T variant, we meanwhile sequenced remaining encoding exons of TET2 (including exon 3~8, 10, 11) in 25 cases with C/T genotype and found that all the C/T genotype patients didn't harbor classic TET2 mutations. Our results showed that the C/T genotype was enriched in the MPN group compared to healthy controls (*P* = 0.01; Odd Ratio (OR) = 2.361; 95% Confidence Interval (CI) = 1.206–4.624), suggesting that TET2 rs3733609 C/T genotype was associated with a predisposition to sporadic MPNs.

**Table 1:** TET2 rs3733609 genotype frequency in MPN patients and controls

Case polulation	Genotype frequency (%)		Odd ratio (95%CI)	P^*^
	T/T	C/T		
MPN (*n* = 181)	86.19	13.81		
Healthy controls (*n*= 236)	93.65	6.35	2.361 (1.206–4.624)	0.01

* *P* based on chi-square test.

### Clinical features of MPN patients with rs3733609 C/T genotype

Our results have shown that TET2 rs3733609 C/T genotype is a risk factor for the development of MPN. Whether MPN cases with rs3733609 C/T genotype exhibited a different clinical features and thrombotic complication from those of MPN with T/T genotype is also the concerned problems. We compared the hematological parameters and clinical characteristics at newly diagnosed 181 MPN patients with JAK2V617F-positive. According to the rs3733609 genotype stratification, there were evident difference on clinical phenotypes and hematological parameters between two groups (Table 2). Our results showed that the leukocyte count, platlet numbers and hemoglobin levels were higher in MPN patients with C/T genotype than those with T/T genotype (*P* < 0.001, *P* = 0.042 and *P* = 0.019, respectively). Compared to T/T genotype, the MPN patients with C/T genotype had higher proliferative activity, which was defined as the hyperplasia degree of bone marrow (*P* = 0.005). Meanwhile, thrombotic events and hepatosplenomegaly were more common in C/T genotype (*P* < 0.01 for both). However, no significant differences were observed in age, sex and cardiovascular risk factors such as tobacco use, hypertension, or diabetes mellitus between the two genotypes.

**Table 2:** Clinical and laboratory features of MPN cases with JAK2V617F-positive stratified according to TET2 rs3733609 genotype

	C/T genotype	T/T genotype	*P* value^*^
Number of patients (M:F)	25 (14:11)	156 (79:77)	0.619
Age (years, median and range)	58 (22–73)	59 (28–90)	0.504
WBC (× 10^9^/L, mean ± SD)	20.8 ± 6.4	13.8 ± 5.4	< 0.001
Hb (g/L, mean ± SD)	176.1 ± 47.9	154.2 ± 39.7	0.019
Platelet (× 10^9^/L, mean ± SD)	835.2 ± 310.8	563.1 ± 228.6	0.042
Bone marrow hyperplasis	23/25 (92.0%)	99/156 (63.5%)	0.005
Myelofibrosis	2/25 (8.0%)	18/156 (11.5%)	0.857
Splenomegaly	16/25 (64.0%)	53/156 (33.97%)	0.004
Hepatomegaly	9/25 (36.0%)	15/156 (9.62%)	0.001
CV risk factors	12/25 (48.0%)	66/130 (42.31%)	0.594
Thrombotic event	13/25 (52.0%)	30/156 (19.23%)	< 0.001

* *P* based on chi-square test or student's *t*-test (two-tailed). SD standard deviation, CV risk factor; cardiovascular risk factors including tobacco use, hypertension, or diabetes mellitus.

### The evaluation of risk factors for thrombosis in JAK2V617F-positive MPN

In 181 MPN patients with JAK2V617F mutation, forty-three cases (23.75%) had thromboembolic events. Among them, 31 cases were arterial embolism (17.13%) and 12 cases were vein thrombosis (6.63%). Since the occurrence of thrombotic complication was higher in MPN patients with rs3733609 C/T genotype than those with T/T genotype, we analyzed the risk factors of thrombosis using univariate analysis and then definited the thrombotic risk factors from a shortlist of seven candidates comprising rs3733609 C/T genotype, sex, age, WBC count, hemoglobin levels, platelet numbers and cardiovascular risk factors(including tobacco use, hypertension, or diabetes mellitus) with reference to related literatures [24]. The univariate analysis results showed that the age more than 60 years (*P* < 0.001), rs3733609 C/T genotype (*P* < 0.001), WBC count ≥ 10 × 10^9^, (*P* < 0.001) and cardiovascular risk factors (*P* < 0.001) were correlated with thrombotic complications; while the sex, hemoglobin levels and platelet numbers were not risk factors for thrombotic events (*P* > 0.05) (Table 3). Subsequently, a logistic regression analysis was applied based on the univariate analysis. The results indicated that advanced age (≥ 60 years vs < 60 years: Hazard Ratio (HR) = 2.9; 95% Confidence Interval (CI) = 1.167–7.202; *P* = 0.022); rs3733609 C/T genotype (C/T genotype vs T/T genotype: HR = 5.011; 95% CI = 1.596–15.735; *P* = 0.006); increased WBC count (≥ 10 × 10^9^/L vs < 10 × 10^9^/L: HR = 5.144; 95% CI = 1.623–16.302; *P* = 0.005) and cardiovascular risk factors (presence vs absence: HR = 3.808; 95% CI = 1.432–10.126; *P* = 0.007) were independent risk factors of thrombosis respectively (Table 4), suggesting that MPN patients with rs3733609 C/T genotype, advanced age, higher white cells count or accompanied cardiovascular risk factors may benefit from the anticipated prevention of thrombo-embolism.

**Table 3:** Univariate analysis of risk factors for thrombosis in JAK2V617F-positive MPNs

	Thrombotic group	Control group	*P* value^*^
Number of patients (M:F)	43 (26:17)	138 (67:71)	0.172
Age (≥ 60 years)	31/43 (72.1%)	43/138 (31.16%)	< 0.001
CV risk factors	36/43 (83.72%)	42/138 (30.43%)	< 0.001
rs3733609 C/T genotype	13/43 (30.2%)	12/138 (8.7%)	< 0.001
WBC (≥ 10 × 10^9^/L)	40/43 (93.02%)	61/138 (44.2%)	< 0.001
Hb (g/L)			
< 160	24	67	
160–180	5	16	
> 180	14	55	0.374
Platelet (× 10^9^/L)			
< 300	5	22	
300–600	17	56	
> 600	21	60	0.454

* *P* based on chi-square test or the Mann-Whitney *U*-test. CV risk factor; cardiovascular risk factors including tobacco use, hypertension, or diabetes mellitus.

**Table 4:** logistic regression analysis of risk factors for thrombosis in JAK2V617F-positive MPN

	B	S.E	Wals	df	Sig.	Exp (B)	95% C I
Age ≥ 60 years	1.065	0.464	5.260	1	0.022	2.900	1.167–7.202
WBC ≥ 10 × 10^9^/L	1.683	0.589	7.745	1	0.005	5.144	1.632–16.302
rs3733609 C/T genotype	1.612	0.584	7.620	1	0.006	5.011	1.596–15.735
CV risk factors	1.337	0.499	7.184	1	0.007	3.808	1.432–10.126

### With attenuated TET2 mRNA transcript in MPN with rs3733609 SNP

**Figure 2:**
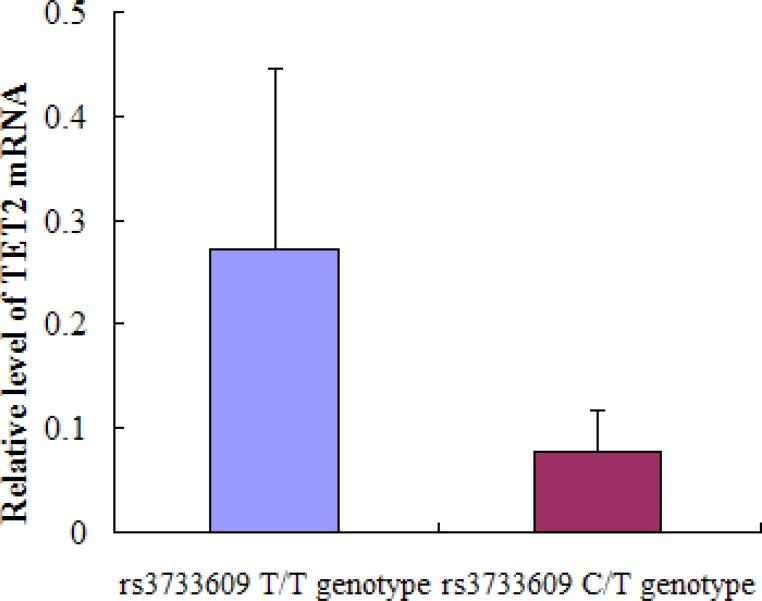
The mRNA expression levels for TET2 gene in bone marrow mononuclear cells between C/T and T/T genotype MPN patients Compared with T/T genotype, TET2 mRNA levels were markedly decreased by 71.2% in rs3733609 C/T genotype group (*P* = 0.006).

The rs3733609 C/T genotype was not only a predisposition factor of MPN, but also correlated to the clinical features or complication of MPN to some extent. To determine the underlying mechanism of rs3733609 C/T genotype-mediated changes, we compared the levels of TET2 mRNA using real-time quantitative PCR between JAK2V617F positive MPN cases with C/T genotype (*n* = 18) and T/T genotype (*n* = 35). The results demonstrated that TET2 mRNA levels were decreased by 71.2% in rs3733609 C/T group in comparison to T/T group (*P* = 0.006, Figure 2), suggesting that rs3733609 C/T genotype down-regulated the transcription of TET2 mRNA.

### rs3733609 C/T genotype interferes with the interaction between C/EBPA and TET2 and down-regulates the transcription of TET2 mRNA

**Figure 3:**
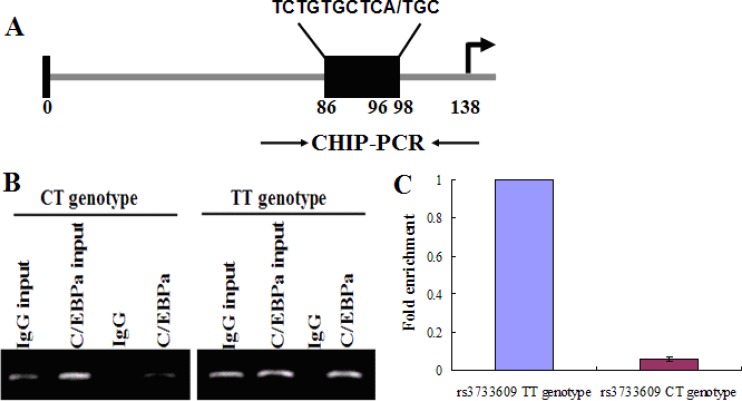
C/EBPA binds to the exon 9 of TET2 (**A**) A schematic diagram for the positions of putative C/EBPA protein binding sites in the exon 9 of TET2. Arrows indicate the regions for PCR primers amplification. (**B**) CHIP assay was performed on human bone marrow mononuclear cells (BMMNCs) using C/EBPA antibody or mouse IgG and the immunoprecipitated chromatin DNA was subjected to PCR using primers to amplify the region of biding sites for C/EBPA. (**C**) Precipitated chromatin DNA were subjected to qPCR to amplify this region, and the results showed that the region of +86 to +98 in TET2 exon 9 was enriched 15.7 fold in the C/EBPA chromatin immunoprecipitates in patients with rs3733609 T/T genotype compared with C/T genotype group (*P* < 0.001).

Next, we tried to reveal how rs3733609 C/T genotype causes the low expression of TET2 mRNA. First, we determined which of transcriptional factors probably bond to the TET2 rs3733609 SNP region using Transfac-Patch 7.0 Software prediction. The result showed C/EBPA could bind to the region of TET2 exon 9 from nucleotides +86 to +98 locus (covering the rs3733609 T/T locus, Figure 3A). Then, we validated this prediction by chromatin immunoprecipitation (CHIP) assay using either an anti-C/EBPA antibody or an IgG control. The quantitative PCR (qPCR) were performed for evaluating the levels of DNA fragment in the precipitates containing C/EBPA-binding sequences derived from TET2 exon 9. Our results indicated that C/EBPA may effectively bind to the rs3733609 T/T loci (Figure 3B), and the region from +86 to +98 was enriched 15.7 fold in the C/EBPA chromatin immunoprecipitates in MPN patients with rs3733609 T/T genotype than those with C/T genotype (*P* < 0.001, Figure 3C), suggesting that C/EBPA transcript factor mediated the regulation of TET2 gene expression by binding to TET2 exon 9 domain localized at nucleotide +86 to +98 sequences, and the C/T genotype weakened the binding of C/EBPA at this region and down-regulated transcription of the TET2 mRNA.

### rs3733609 C/T genotype impacts the transcript activity of the TET2 gene

**Figure 4:**
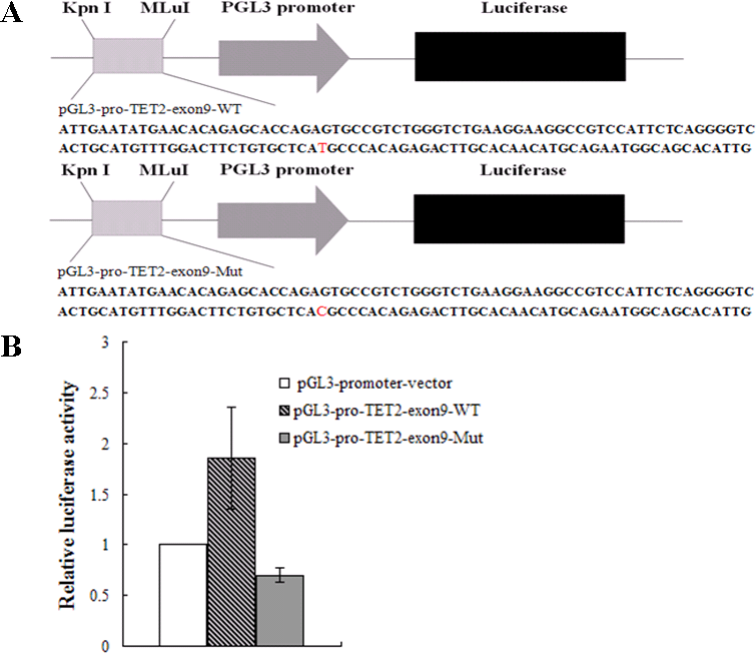
Luciferase activities assay (**A**) A schematic diagram of the reporter constructs containing the sequences of TET2 exon 9. The mutant construct containing identified mutation site (position +96) in the TET2 exon 9 is shown. (**B**) Results are shown as fold change of luciferase activity corresponding to pGL3-promoter vector, the wild vector displayed the stronger luciferase activity than the pGL3-promoter-vector (*P* = 0.014); the luciferase activity of mutant vector was dramatically reduced to 0.38 fold in transfected HEL cells than that of the wild-type vector (*P* = 0.009). Values are means ± SD of triplet data from 3 different experiments.

To investigate the effect of the TET2 gene with rs3733609 C/T genotype on transcript activity, we cloned the exon 9 fragment of TET2 gene with both rs3733609 T/T or C/T genotype sequences (T/T genotype vector: pGL3-pro-TET2-exon9-WT; C/T genotype vector: pGL3-pro-TET2-exon9-Mut96) into pGL3-promoter-vector (Figure 4A) and conducted the luciferase reporter activity assay in HEL cells(ATCC:TIB-180). Before this experiment, we had screened the mutant status of JAK2V617F and TET2 in HEL cells by PCR sequencing. The results confirmed that HEL cells harbored JAK2V617F homozygous mutation, and no any mutation was found in all exons of TET2. The pGL3-promoter-vector, T/T genotype or C/T genotype vector was electrotransfected into HEL cells respectively. 48 hours after transfection, the three reporter vectors displayed different significantly luciferase activity (Figure 4B). The T/T genotype vector exhibited the stronger luciferase activity than the pGL3-promoter-vector (*P* = 0.014). Compared with the T/T genotype vector, the luciferase activity of C/T genotype vector of TET2 gene was reduced (*P* = 0.009) on transfected HEL cells, suggesting that the rs3733609 T locus in TET2 exon 9 was a critical positive regulatory element, and the T to C variant at this position impeded its transcriptional activity.

### rs3733609 variant contributes to erythroid hematopoiesis on MPN patients-BFU-E assay results

**Figure 5:**
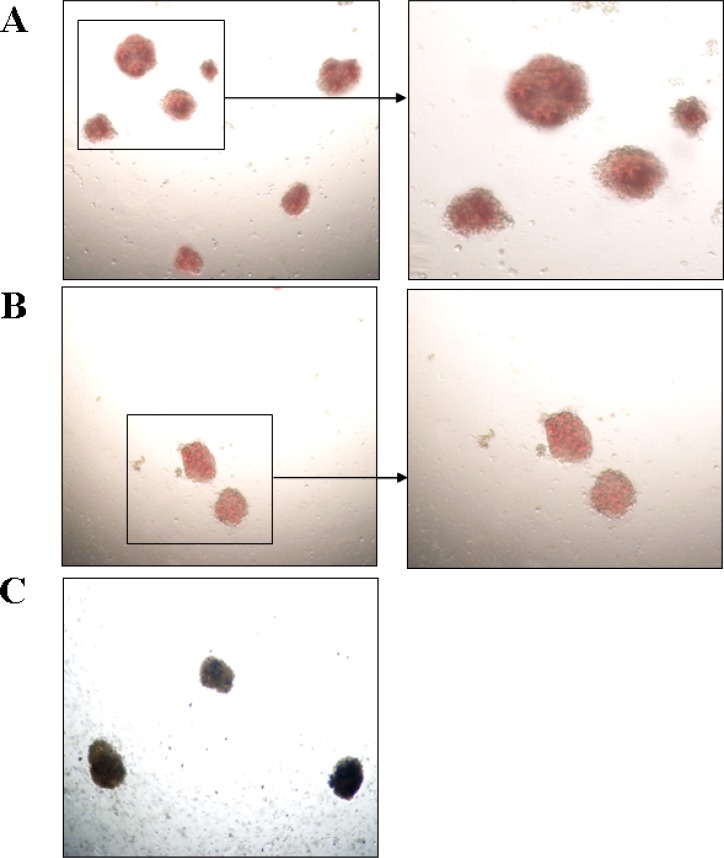
Photomicrographs of BFU-E colonies *in vitro* (Left: magnification 40×; Right: magnification 100×) (**A**) The BFU-E colonies derived from the bone marrow mononuclear cells with C/T genotype. (**B**) The BFU-E colonies derived from the bone marrow mononuclear cells with T/T genotype. (**C**) Colonies were stained by benzidine staining. Blue-black colonies were BFU-E colonies. As shown in Figure A and B, the colony numbers and volume from C/T genotype patients were more and larger than that of T/T genotype.

To clarify the effect of rs3733609 C/T genotype on the erythroid hematopoiesis *in vitro*, we performed burst-forming unit-erythroid (BFU-E) assays on BMMNCs from 39 cases of JAK2V617F positive MPN patients with (*n* = 16) or without rs3733609 C/T genotype (*n* = 23). The results showed that the percentages of BFU-E formation in C/T genotype was higher than that in T/T genotypes (*P* = 0.005) (Table 5). Moreover, compared with T/T genotype, leukocyte count, platelet number and hemoglobin levels were higher in C/T genotype (*P* < 0.001, respectively) (Table 5). We also observed that both colony numbers and colony volumes were increased by 0.6 fold and 1 fold respectively in C/T genotype than those in T/T genotype (*P* = 0.001) (Table 5; Figure 5A, 5B). Altogether, the observation suggested that the hematopoietic stem/progenitor cells of the MPN patients with C/T genotype possessed a facilitating proliferative potential on erythroid hematopoiesis.

**Table 5:** The impact of rs3733609 C/T genotype on the BFU-E colonies formation

	Genotype		*P* value^*^
	C/T	T/T	
Number of patients (M:F)	16 (9:7)	23 (12:11)	0.802
Age (years, mean ± SD)	55.3 ± 13.8	53.5 ± 10.7	0.264
WBC(10^9^/L, mean ± SD)	23.03 ± 4.25	17.72 ± 6.11	< 0.001
Hb (g/L, mean ± SD)	200.7 ± 37.4	159.2 ± 32.8	< 0.001
Platelet(10^9^/L, mean ± SD)	703.3 ± 175.9	524.1 ± 192.3	< 0.001
BFU-E (%)	16/16 (100%)	14/23 (60.9%)	0.005
Number of BFU-E (mean ± SD)	32.06 ± 11.4	20.36 ± 5.54	0.001

* *P* based on chi-square test or student's *t*-test (two-tailed). SD standard deviation.

## DISCUSSION

In MPN, TET2 mutations have been associated with an increased risk of leukemic transformation and short survival [23]. Interestingly, TET2 mutations have been found in healthy elder females who display clonal hematopoiesis in the absence of any blood count parameter abnormalties [25], suggesting TET2 mutations may confer a clonal advantage at the level of the HSC. Our previous results have indicated that deficiency of TET2 function results in unfavorable overall survival of MDS/MPN [26]. In the present study, we first noted that the frequency of the TET2 rs3733609 C/T genotype was higher in sporadic MPN patients than in control populations (OR = 2.361; *P* = 0.01), suggesting that rs3733609 C is a MPN predisposition allele.

Given these findings, we then wished to determine whether the MPN cases with rs3733609 C/T genotype display different characteristics of clinical and laboratory from those with T/T genotype. To do so, we compared and analysed some variables by univariate and multivariate analysis. Our results showed that compared with T/T genotype, MPN cases with rs3733609 C/T genotype had elevated leukocyte and platelet counts, increased hemoglobin levels; and an higher incidences of hepatosplenomegaly and thrombotic events, indicating C/T genotype contributes to myeloid hematopoiesis of MPN (Table 5; Figure 5). In addition, the colony numbers and volumes derived from BMMNCs in C/T genotype increased by 0.6 and 1 times respectively than those in T/T genotype. This proliferative potential aggravates the clinical symptoms or complications of MPN.

We also attempted to reveal the mechanisms of rs3733609 C/T genotype on the pathogenesis and the susceptibility of MPN. We analyzed BMMNCs samples from MPN patients with or without rs3733609 C/T genotype using a real-time quantitative-PCR assay, which showed that the expression of TET2 mRNA in rs3733609 C/T genotype decreased by 71.2% than that in T/T genotypes (*P* = 0.006). To further confirm this change, we performed luciferase activity assay, and obtained a consistent results with RT-PCR in that the transcriptional activities produced by pGL3-pro-TET2-exon9-Mut96 were lower than the wild-type construct (*P* = 0.009), indicating the rs3733609 T/T locus was involved in the basal activation of the transcription of TET2.

In hematopoiesis, C/EBPA(CAAT/enhancer binding proteins alpha) is a key factor in driving the development of myeloid cells through interacting with a variety of factors(including c-Myc, PU.1 and microRNAs), and mutations of C/EBPA have been observed in about 10% of patients with acute myeloid leukemia (AML) [27, 28]. As an important transcript regulator, it is possible that C/EBPA or its binding sites are involved in the basal activation of the transcription of TET2. According to the prediction of bioinformatics and CHIP results, We found that C/EBPA is able to bind with TET2 DNA at the locus of exon 9 from nucleotides +86 to +98 sequences, and rs3733609 C/T genotype can attenuate the binding of C/EBPA and TET2, resulting in the down-regulation of TET2 transcription. Therefore, the interaction between the TET2 DNA and C/EBPA contributes obviously to the transcription of TET2, and the rs3733609 T/T locus is a positive regulatory element for controlling the basal transcriptional activities of TET2.

In conclusion, we report a novel germline variant in TET2 that associates with JAK2V617F-positive MPN. The risk regulated by rs3733609 C/T variant is higher (OR = 2.361) than the reported JAK2 germline signal rs1034072 A (OR = 1.85). Moreover the variant also exerts effect on myeloid hematopoiesis by promoting proliferation of myeloid progenitor cells, which is related to the decreased transcript activity of TET2. and, this study characterizes the mechanism by which rs3733609 C/T variant escapes from the positive regulation of C/EBPA on TET2 transcription.

## MATERIALS AND METHODS

### Patients and controls

One hundred and eighty-one consecutive MPN patients (85 PV, 78 ET, 18 PMF), diagnosed between 2005 and 2015, were enrolled into the study after carefully revising all of diagnosis according to the criteria of World Health Organization classification of MPN [29]. The control group consisted of 236 unrelated local volunteers. Written consent regarding genetic testing was obtained from all of patients and controls. This study was approved by the Ethics Committee of The Second Xiang-Ya Hospital, Central South University and conducted in accordance with the World Medical Association Declaration of Helsinki.

### Definitions of thrombosis events

Thrombotic events were defined as arterial or venous events occurring prior to diagnosis or at any time post diagnosis of MPNs. For arterial events, cardiovascular events included myocardial infarction and unstable angina, which were confirmed by an elevated troponin I level or with electrocardiogram changes. Cerebrovascular events included radiologically verified cerebrovascular accidents or transient ischaemic attacks with consistent history. Peripheral vascular events included new onset of intermittent claudication or acute limb ischaemia. Venous thromboembolic events included radiologically or ultrasonically confirmed portal vein, abdominal vein and deep venous thrombosis with elevated D-D levels.

### Molecular analysis for JAK2V617F mutation and TET2 rs3733609 SNP

Genomic DNA was obtained from peripheral blood or bone marrow using a commercially available BloodGen Kit (ConWin, China). The JAK2 V617F mutational status was analyzed in all MPN patients by a allele specific PCR method, as previously described [30]. A 475 bp PCR product containing rs3733609 loci, where TET2 exon 9 was included, was amplified from 181 cases of MPN with JAK2V617F-positive and 236 healthy controls. The sequences of primers for PCR amplification were: forward primer: 5′-CCATGTCAAGATATTTGCTC-3′; reverse primer: 5′-TTTGCTCCTCATTTGCCTTC -3′. The PCR products from each subject were purified and sequenced using ABI 3730 XL Genetic Analyzer by BioSune Biotechnology Company, China.

### Effect of rs3733609 variation on TET2 gene transcription

To assess whether TET2 mRNA expression was influenced by rs3733609 SNP, real-time quantitative PCR was performed for evaluating the expressions of TET2 mRNA. 35 of MPN with rs3733609 T/T genotype and 18 of MPN with rs3733609 C/T genetype were compared for TET2 mRNA expressive abundance. In brief, bone marrow (5 mL) mononuclear cells (BMMNCs) were isolated by Ficoll centrifugation; total cellular RNA from 1 × 10^6^ cells/mL was extracted by Trizol reagents (Invitrogen). Random primers and M-MLV reverse transcriptase (TaKaRa) were used to generate cDNA. Real-time quantitative PCR was carried out on a LightCycler 96 (Roche, Switzerland) using SYBR^®^ Premix Ex Taq TM II Kit (TaKaRa). Primers sequences were as follows: Human TET2-specific primers: forward primer: 5′-CTATTGCTAAGTGGGTGGTTCG-3′; reverse primer: 5′-GCCGTATTTCCTCAGCGTCTC -3′. Human β-actin amplifation was designed as internal control. The PCR reactive system comprised cDNA template 2 uL, 2xSYBR mix 10 uL, forward or reverse primers 10 uM.

### Chromatin immunoprecipitation (CHIP) assay

The prediction of transcriptional factor binding site for TET2 rs3733609 SNP was performed with Transfac-Patch 7.0 Software (http://www.gene-regulation.com/cgi-bin/pub/programs/patch/bin/patch.cgi), and the predicted results indicated that rs3733609 (T/T genotype) loci could bind to C/EBPA protein domain (Figure 3A). In order to document the binding, the Chromatin Immunoprecipitation (CHIP) assay was carried out according to the instructions of EZ ChIP^™^ Kit (Cat#17–371, Millipore). Briefly, BMMNCs (1 × 10^7^/ml) from 16 of MPN patients with rs3733609 C/T genotype and 20 of MPN cases with rs3733609 T/T genotype were crosslinked respectively for 10 minutes in 1% formaldehyde solution. Then, crosslinked chromatins were prepared and sonicated to an average size of 500 bp. Chromatin-protein complexes were immunoprecipitated with antibody specific to C/EBPA (sc-61X; Santa Cruz Biotechnology) or normal mouse IgG at 4°C for overnight. Input DNA was precipitated without antibody. After reversal of cross-linking, the region of interested was amplified from precipitated samples by real-time quantitative PCR. For the amplification of TET2 gene fraction, the primers sequences were as follows: forward primer: 5′-CCGTCTGGGTCTGAAGGAAG-3′; reverse primer: 5′-CTGTCCTCAGCCCAACTTACC-3′. Each sample was assayed in triplicate, and the amount of precipitated DNA was calculated as the percentage of input sample.

### Reporter gene construct and luciferase activity assays

To further testify whether rs3733609 C/T genotype is able to affect the transcriptional activity of the TET2 gene, a corresponding reporter gene plasmid was constructed as follows: 1). pGL3-promoter vector (empty vector, Promega, USA), containing the SV40 promoter of driving the firefly luciferase gene; 2). pGL3-pro-TET2-exon9-wt, containing the luciferase reporter gene and carrying 138 bp of TET2 exon 9 (rs3733609 T/T genotype); 3). pGL3-pro-TET2-exon9-Mut96 (rs3733609 C/T genotype), containing the TET2 exon 9 c.138 + 96T > C variation. The exon 9 and its variant form (+96 T > C variation) were respectively inserted into the junction region between Kpn I and MLuI restriction locus (Figure 4A). The constructions of the reporter gene were performed by TaKaRa Company (Dalian, China). Briefly, TET2 exon 9 wild and variant sequences were directly synthesized and subcloned into the pGL3-promoter vector using DNA Ligation Kit (TaKaRa, Japan). Transient transfection of pGL3 promoter-based constructs in HEL cells was performed using the Amaxa Nucleofector Technology (Lonza Cologne, Cologne, Germany). Basic procedure included: 1 × 10^6^ HEL cells were electroporated in 100 ul Ingenio Electroporation Solution (Mirus Bio, Madison, WI) containing 5 ug of the pGL3 promoter construct and 0.5 ug pRL-TK plasmid using T016 electroporation program [31]. At 48 h after electroporation, the cells were harvested in passive lysis buffer, and luciferase activity was measured by means of a dual luciferase assay (Promega). Firefly luciferase activities were normalized to Renilla luciferase controls. The experiments were performed in triplicate in three independent experiments. All results were expressed as fold increases or decreases over the levels produced by cell transfection with the pGL3-promoter vector (empty vector).

### Erythroid burst-forming unit-colony formation assay

To compare the hematopoietic potential between MPN patients with or without rs3733609 C/T variant, the colony formation experiment for burst-forming unit-erythroid (BFU-E) was performed. In briefly, 1 × 10^5^ BMMCs from MPN patients with rs3733609 C/T variant were plated in 1 ml of RPMI-1640 methylcellulose culture without any cytokines. Meanwhile, the BMMCs from MPN patients with T/T genotype were used as the control. The culture medium consist of 0.8% methylcellulose (Stem Cell Technologies, Vancouver, Canada), 1% deionised bovine serum albumin (BSA), 30% FCS and 2-β-mercaptoethanol (10^−4^ M). Culture dishes were incubated at 37°C for 14 d in a highly humidified atmosphere with 5% CO_2_. The colonies were identified by benzidine staining (Figure 5C) and enumerated using an inverted microscope. The BFU-E colony was defined as a colony in which colony size reached more than 50 cells and displayed red-tined cytoplasm, or positive reaction for benzidine staining on 14th day [32].

### Statistical analysis

The frequencies of the genotypes between MPN cases and controls were compared by Pearson's Chi-square test or Fisher's exact test when required. The data was expressed as the means ± SD. Univariate analysis was treated using Student's *t*-test (two-tailed) or the Mann-Whitney *U*-test. Logistic regression analysis was used for multivariate analysis. SPSS Version 19.0 (SPSS, Chicago, IL) was used for all statistical tests. In all tests, *P* < 0.05 was defined as statistically significant.
